# Functional evaluation of rat hearts transplanted after preservation in a high-pressure gaseous mixture of carbon monoxide and oxygen

**DOI:** 10.1038/srep32120

**Published:** 2016-08-26

**Authors:** Naoyuki Hatayama, Masayuki Inubushi, Munekazu Naito, Shuichi Hirai, Yong-Nan Jin, Atsushi B. Tsuji, Kunihiro Seki, Masahiro Itoh, Tsuneo Saga, Xiao-Kang Li

**Affiliations:** 1 Department of Anatomy, Tokyo Medical University, Tokyo, Japan; 2 Department of Anatomy, Aichi Medical University, 1-1 Yazakokarimata, Nagakute-city, Aichi Pref., 480-1195, Japan; 3 Molecular Imaging Center, National Institute of Radiological Sciences, Chiba, Japan; 4 Department of Nuclear Medicine, Kawasaki Medical School, Kurashiki, Japan; 5 High Altitude Pathology Institute, La Paz, Bolivia; 6 National Research Institute for Child Health and Development, Tokyo, Japan

## Abstract

We recently succeeded in resuscitating an extracted rat heart following 24–48 hours of preservation in a high-pressure gaseous mixture of carbon monoxide (CO) and oxygen (O_2_). This study aimed to examine the function of rat hearts transplanted after being preserved in the high-pressure CO and O_2_ gas mixture. The hearts of donor rats were preserved in a chamber filled with CO and O_2_ under high pressure for 24 h (CO24h) or 48 h at 4 °C. For the positive control (PC) group, hearts immediately extracted from donor rats were used for transplantation. The preserved hearts were transplanted into recipient rats by heterotopic cervical heart transplantation. CO toxicity does not affect the grafts or the recipients. Light microscopy and [^18^F]-fluorodeoxyglucose positron emission tomography revealed that there were no significant differences in the size of the myocardial infarction or apoptosis of myocardial cells in post-transplant hearts between the PC and CO24h groups. Furthermore, at 100 days after the transplantation, the heart rate, weight and histological staining of the post-transplanted hearts did not differ significantly between the PC and CO24h groups. These results indicate that the function of rat hearts is well preserved after 24 hours of high-pressure preservation in a CO and O_2_ gas mixture. Therefore, high-pressure preservation in a gas mixture can be a useful method for organ preservation.

Currently, the preservation time for clinically extracted hearts prior to transplantation is 4–6 hours^1^. Typically, organs are preserved by immersion in a low-temperature (0–4 °C) stock solution after the removal of blood components from the vascular inflow before or after extraction (simple immersion method)^2^,^3^. Stock solutions, such as the University of Wisconsin (UW) and histidine–tryptophan–ketoglutarate solutions, and/or machine perfusion (persistent perfusion method) have been developed to improve the quality of the preserved organs and to extend the acceptable time limits for transplantation^4^,^5^,^6^.

Carbon monoxide (CO) can affect mitochondria and can control metabolism by exerting vasoactive, anti-proliferative, anti-oxidant, anti-inflammatory, and anti-apoptotic effects^7^. The beneficial effects of CO can contribute substantially to cell protection and prevent ischemia–reperfusion injury (IRI) to some degree following transplantation^7^,^8^,^9^. IRI of a transplanted small intestine from a donor rat was significantly alleviated when the small intestine had been preserved for 6 hours at 0–4 °C before transplantation into the recipient rat and low-concentration CO was directly administered by inhalation^9^. Furthermore, it has been reported that the degree of IRI following transplantation was alleviated when pig kidneys and/or rat livers were preserved in a stock solution with a low concentration of CO^10^,^11^.

Hyperbaric O_2_ treatment (high-pressure O_2_) reduces cell death by decreasing the expression of hypoxia-inducible factor-1alpha, p53, caspase-9 and caspase-3^12^,^13^ and by mitigating reperfusion failure-induced neutrophil adhesion by blocking CD18 polarization through a nitric oxide mechanism^14^. However, hyperbaric O_2_ treatment also damages lung function, which is related to cellular dysfunction. Clinically, hyperbaric O_2_ treatment is used in very limited conditions and after heart surgery, and extended high concentrations of inhaled O_2_ are usually avoided^15^. In fact, our previous study revealed that rat hearts could not be resuscitated after 48 hours of preservation under a condition of high-pressure O_2_ (helium (He) = 4000 hPa + PO_2_ = 3000 hPa)^12^. In addition, a condition of high-pressure CO (CO = 4000 hPa + PHe = 3000 hPa) also could not preserve the rat hearts for 48 hours (data not shown). Thus, it seems that a combination of CO and O2 is needed for organ preservation.

Recently, we developed a high-pressure preservation method using a mixture of CO and oxygen (O_2_) gases and succeeded in resuscitating extracted rat hearts following 24–48 hours of preservation under a condition of 7000 hPa {Partial Pressure (P) CO = 4000 hPa + PO_2_ = 3000 hPa}^12^. This preservation method is different from those used conventionally; in our method, organs are preserved in a high-pressure gas rather than in a solution (Fig. 1). The humidity in the chamber is maintained at approximately 100%, and the preserved organ is not severely dehydrated. This preservation method can efficiently increase the effects of the mixture of the CO and O_2_ gases. There is currently no detailed information regarding the effects of high-pressure preservation and CO toxicity on heart function in organs preserved in a mixture of CO and O_2_ gases. Therefore, this study aimed to examine the function of rat hearts transplanted after high-pressure preservation in a mixture of CO and O_2_ gases.

## Results

### CO concentration in the handling preservation chamber

**Figure 2:**
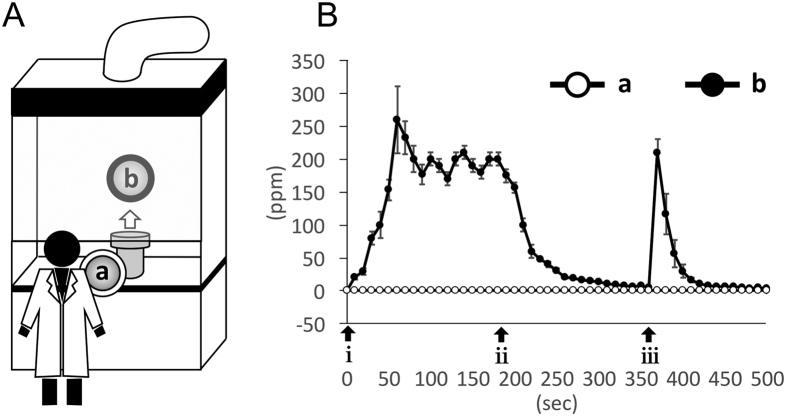
Measurement of the CO concentration in the handling preservation chamber. (**A**) The measurement location of the CO gas after opening the valve of the chamber. “a”: human working area 30 cm from the chamber. “b”: 10 cm directly above the chamber. (**B**) CO concentration at point “a” and “b”. i: opening the valve of the chamber after preservation. ii: pressure gauge zero. iii: opening the cover of the chamber to remove the preserved hearts.

When filling the gases into the chamber, CO gas was not detected directly above the chamber. When opening the valve of the chamber inside the fume food, CO gas was detected on average 260 ppm at approximately 60 sec, decreased at approximately 180 sec, and then was barely detectable at approximately 240 sec. Meanwhile, CO gas was not detected at any time outside the fume hood (Fig. 2). The CO gas that was detected directly above the preserved hearts taken from the chamber was 0 ppm.

### Short-term evaluation (post-transplantation at 90 min)

#### Carboxyhemoglobin (COHb) levels in the recipient’s arterial blood

There was no significant difference between the PC (0.26 ± 0.11%) and CO24h groups (0.22 ± 0.08%) at 90 min after heart transplantation. At 24 h after heart transplantation, the same results were observed (PC: 0.31 ± 0.7%, CO24h: 0.28 ± 0.12%).

#### Myocardial infarct area via TTC staining

**Figure 3:**
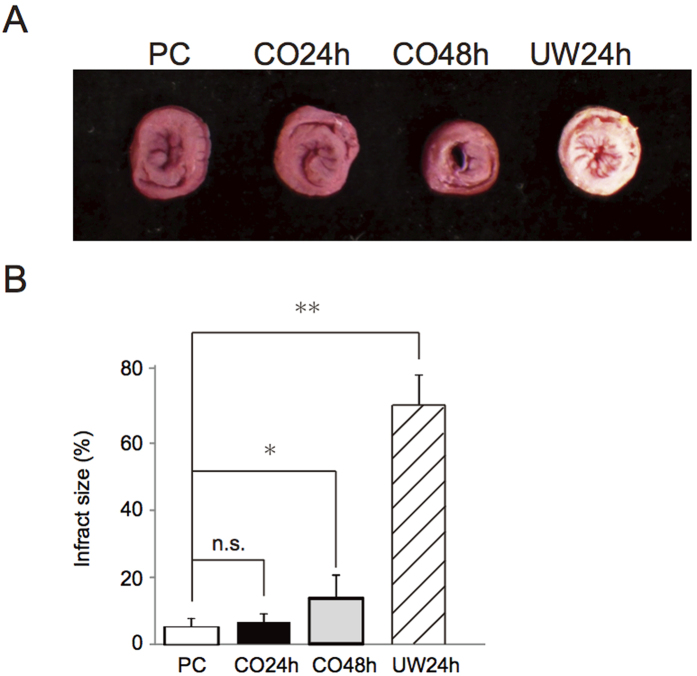
Assessment of myocardial infarct size using 2,3,5-triphenyltetrazolium chloride (TTC) staining in post-transplanted donor hearts at 90 minutes. (**A**) Representative mid-myocardial cross sections of TTC-stained hearts for the 4 groups. Red-stained areas indicate viable tissue and white areas indicate infarct tissue. (**B**) Myocardial infarct size was assessed in the hearts from the 4 groups. P < 0.05 was considered statistically significant. n.s.: not significant, *P < 0.01, **P < 0.05.

PC and CO24 were almost never detected in the infraction area. Also, there was no significant difference observed between both groups (PC: 3.9% ± 1.1, CO24: 4.0% ± 1.7). CO48 was detected in a few infraction areas (13.4% ± 4.5, P < 0.01 compared with control), whereas UW24h was detected frequently in the infraction areas (69.4% ± 8.5) (Fig. 3).

#### The structure and apoptosis of myocardium by histological analysis

**Figure 4:**
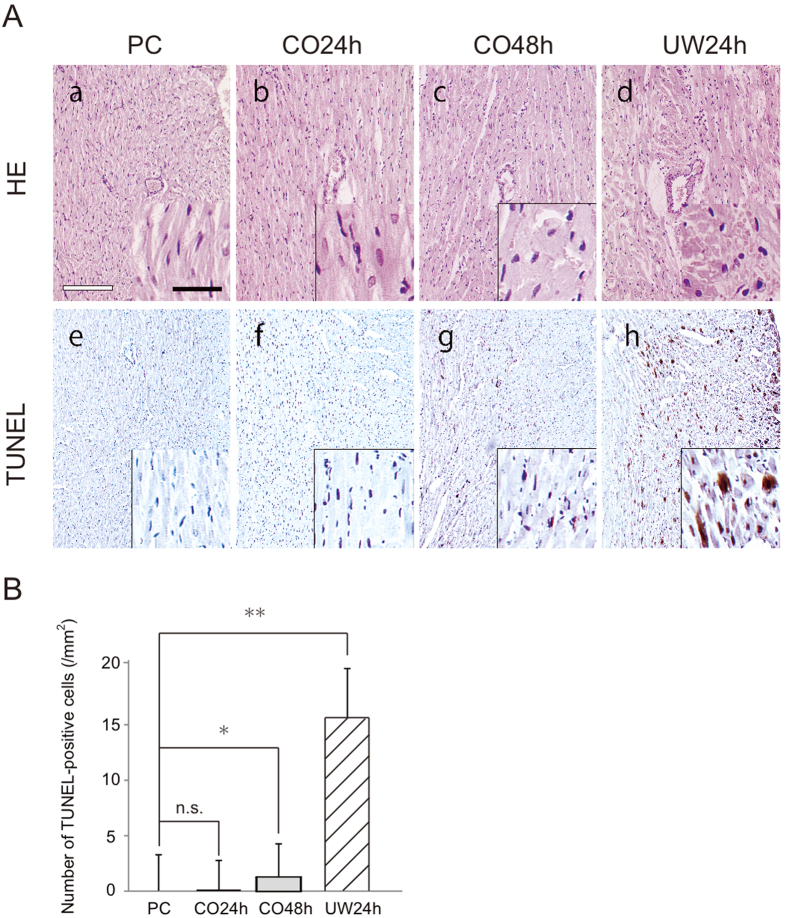
Light microscopy analysis of the post-transplanted donor hearts at 90 minutes. (**A**) Hematoxylin and eosin staining (a: PC, b: CO24h, c: CO48h, d: UW24h) and TdT mediated dUTP nick end labeling (e: PC, f: CO24h, g: CO48h, h: UW24h). (**B**) Quantitation of the number of TUNEL-positive cells. Data from each group are expressed as the means ± standard deviations. P < 0.05 was considered statistically significant. n.s.: not significant, *P < 0.01, **P < 0.05, White bar = 250 μm, Black bar = 50 μm.

Light microscopy analysis showed that the myocardium of the PC, CO24h, and CO48h groups at 90 min after transplantation maintained almost normal tissue structure and shape, and it was difficult to find the differences between them. The UW24h group showed acute IRI, as characterized by hemorrhage and some neutrophil infiltration. The myocardial fibers in the UW24h group were partially ruptured and lysed (Fig. 4A). Using immunohistochemistry analysis, TUNEL-positive myocardial cells were seldom detected in PC (0.15 ± 0.3) and CO24h (0.25 ± 0.5) hearts and were occasionally detected in CO48h (1.25 ± 1.9) hearts, whereas many TUNEL-positive myocardial cells could be observed in UW24h (14.2 ± 8.2) hearts (Fig. 4A). The number of TUNEL-positive myocardial cells in the CO24h group showed no significant change compared with the PC group (P > 0.05), but the number was significantly lower than in the CO48h group (P < 0.01) (Fig. 4B). Air24, Air48, UW48, EF24 and EF48 were unable to be compared because the state of the resuscitation was poor. Meanwhile, there was no significant difference detected in the histological evaluations of the hearts before transplantation when compared with each group.

#### Glucose metabolism in the myocardium

**Figure 5:**
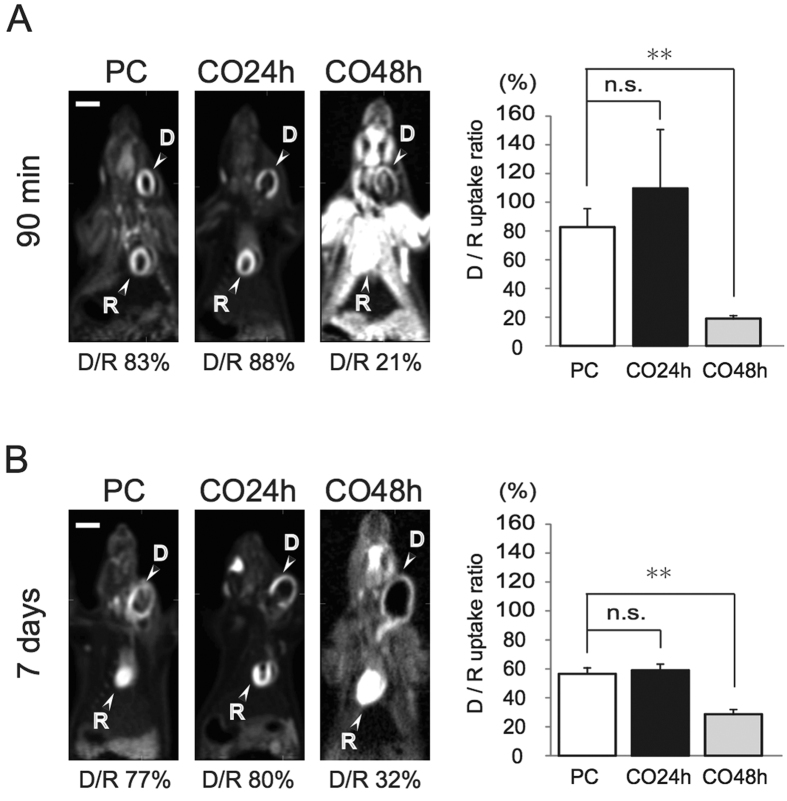
[^18^F]-fluorodeoxyglucose positron emission tomography analysis of the transplanted hearts. (**A**) Post-transplanted donor hearts were imaged at 90 minutes. (**B**) Post-transplanted donor hearts were imaged at 7 days. A semiquantitative evaluation was performed by calculating the maximum accumulation of [^18^F]-FDG in the recipient hearts relative to that of the donor hearts. Data from each group are expressed as the means ± standard deviations. The ANOVA/post-hoc test was employed for statistical analysis on days 0 and 7. P < 0.05 was considered statistically significant. n.s.: not significant, **P < 0.05, D: donor, R: recipient, White bar = 10 mm.

FDG-PET analysis revealed that the maximum [^18^F]-FDG accumulation in the recipients’ hearts relative to that in the donor hearts at 90 min was 80 ± 17% in the PC group, 118 ± 42% in the CO24h group, and 20 ± 2% in the CO48h group (Fig. 5). There was no significant difference between the PC and CO24h groups at 90 min (P > 0.05), whereas [^18^F]-FDG accumulation in the CO48h group was lower than that in the PC group (90 min, P < 0.05). UW24 was not detected because it was unable to be resuscitated.

### Long-term evaluation (at 100 days post-transplantation)

#### Survival rate of transplanted hearts

Revival rates immediately after transplantation and survival rates after 100 days for each group are shown in Table 1. The revival rate was 100% (6/6), whereas the survival rate at 100 days was 83.3% (5/6) in the PC and CO24h groups. In the CO48h group, although the revival rate was 100% (6/6), the survival rate after 100 days was 66.7% (4/6). In the UW24h group, one heart was resuscitated (1/6) but stopped on day 1. In the other groups (UW48h, Air24h, Air48h, EF24h, and EF48h), no post-transplanted hearts were resuscitated (0/6). In these five groups, the transplanted heart was in an undoubtedly poor state, and therefore, did not perform well.

#### Condition of transplanted hearts at post-operative day 100

**Figure 6:**
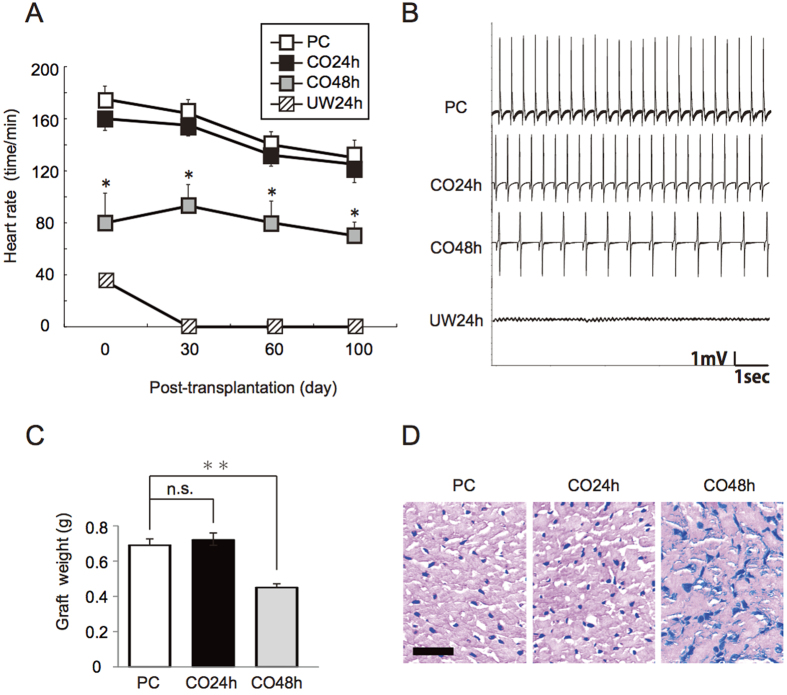
Long-term evaluation of post-transplanted donor hearts at 100 days. (**A**) Time-dependent changes in heart rate were evaluated. (**B**) Electrocardiogram images of the transplanted heart. (**C**) Weight of the transplanted heart. (**D**) Hematoxylin and eosin staining. P < 0.05 was considered statistically significant. n.s.: not significant, *P < 0.01, **P < 0.05, Black bar = 100 μm.

Changes in the heart rates in the post-transplanted hearts on days 0, 30, 60, and 100 are shown in Fig. 6. In the PC and CO24h groups, the heart rate was 160–180 beats/min at 90 min, and it gradually decreased by day 100. On day 100, the heart rates in the PC and CO24h groups were 128 ± 13 beats/min and 132 ± 15 beats/min, respectively. The heart rate did not differ significantly between the PC and CO24h groups on days 0, 30, 60, and 100 (P > 0.05). In the CO48h group, the heart rate was 80 ± 15 beats/min on day 0, but it increased slightly on day 30 and decreased to 70 ± 5 beats/min on day 100. The heart rates in the CO48h group were significantly lower than those in the PC group on days 0, 30, 60, and 100 (P < 0.01) (Fig. 6A). On day 100, electrocardiograms also yielded the same results (Fig. 6B). The heart weights on day 100 in the PC and CO24h groups were 0.70 ± 0.03 g and 0.72 ± 0.04 g, respectively, with no significant difference between the two groups. In the CO48h group, the heart weight on day 100 was 0.42 ± 0.08 g and was significantly lower compared with the PC group (P < 0.01) (Fig. 6C). On day 100, the transplanted hearts in the control and CO24h groups showed mild fibrosis with a few foci of neutrophil infiltration; however, there was no obvious difference in histological findings between these two groups. In the CO48h group, severe fibrosis with some neutrophil infiltration was observed on day 100 (Fig. 6D).

## Discussion

In the present study, to evaluate the function of rat hearts following high-pressure preservation in a mixture of CO and O_2_ gases, we transplanted preserved hearts into recipient rats via heterotopic cervical heart transplantation and performed histopathological and FDG-PET analyses. The results showed no significant differences in the transplanted hearts between the PC and CO24h groups. In the CO-UW48h and UW24h groups, the transplanted hearts failed to function properly. To our knowledge, the present study is the first to evaluate the function of rat hearts following their preservation in a high-pressure mixture of CO and O_2_ gases.

Advanced tissue engineering and regenerative medicine are the current focus of research, with efforts being made to meet the demands of patients who not suitable or eligible for organ transplantation. However, the demand for organ transplantation as a standard therapy continues to increase^16^. One important factor for improving the clinical outcome of transplantation procedures is the quality of the donor organ^5^. Therefore, to improve the performance of transplantation procedures, an effective method of organ preservation is important^5^. However, techniques for donor heart preservation have shown little improvement over the last several decades^17^. To enhance the current quality of donor hearts, researchers continue to experiment with various methods in animals. For example, improvement of the UW solution^18^, a two-layer method using UW^19^, and super cooling with UW^20^ have all been attempted. These attempts have resulted in the stock solution having organ- and/or cell-protective effects because immersion in a stock solution is generally considered important to organ preservation^10^,^11^. However, in our results, only one of six post-transplanted hearts in the UW24h group could be resuscitated. The clinical use of the commonly used UW solution was confirmed to be not suitable for long-term preservation. The post-transplanted hearts in the CO24h and CO48h groups could survive for 100 days after transplantation. These results indicate that a high-pressure preservation in a mixture of CO and O_2_ gases is useful for maintaining the quality of the long-term preserved donor heart.

CO has cell protective effects, which were verified by functional evaluation and the high-pressure preservation method. However, CO toxicity is still a concern. In the present study, while the heart was being preserved, the CO gas remained in the chamber. Because four bolts hermetically sealed the chamber, the chamber can be safely transported. The preserved heart was safely taken from the chamber within approximately 6 min inside the fume hood. Moreover, it became evident that dissolved CO gas in the donor hearts had no effect on the recipient based upon the COHb levels in the recipient’s arterial blood. These results demonstrate that this preservation method is safe for the organ procurement preservation team, transporters of the organ and the recipients. These results also suggest the utility of the high-pressure preservation method for safely preserving the organ for the recipient, which is of clinical importance.

In the present study, histological evaluations were performed after the transplantation to confirm IRI in the preserved hearts. TTC staining of horizontal sections of the ventricle demonstrated that high-pressure preservation reduced myocardial infraction of the whole heart by permeating not only the gas-contacting surface but also the internal heart. Light microscopy analysis showed that the myocardium of the PC, CO24h and CO48h groups at 90 min after transplantation maintained an almost normal tissue structure and shape, and it was difficult to find differences between them. Moreover, few TUNEL-positive myocardial cells could be observed in the CO24h and CO48h groups, whereas many TUNEL-positive myocardial cells could be detected in the UW24h group. These results indicated that cell death in the preserved hearts was suppressed, and IRI in the preserved hearts was reduced by the mixture of high-pressure CO and O_2_ gases. A calculation from previous data according to Henry’s Law reveals that that 0.0945 ml of CO and 0.1716 ml of O_2_ dissolve into 1 ml of H_2_O under a pressure of 7000 hPa (PCO = 4000 hPa + PO_2_ = 3000 hPa)^21^. The current results imply that these doses of the two gases are useful to reduce reperfusion failure in the rat hearts. Under higher pressure, the amount of CO and O_2_ that can be dissolved into H_2_O increases. In this study, the maximum pressure (7000 hPa), which is limited by the capacity of the preservation chamber, was attempted. However, the results did not indicate that this is an optimal condition. Because the higher pressure will damage the preserved hearts, a pressure lower than 7000 hPa might be better. We also need to consider whether there is a more optimal ratio of the two gases for preservation than this ratio. Future investigation of the molecules associated with the CO and O_2_ combination will lead to discovering the optimal conditions for high-pressure preservation with mixed gases. An investigation into the effects that the difference in the pressure and partial pressure ratio of the mixture of CO and O2 gases have on a preserved heart is now planned.

Heterotopic cervical heart transplantations have been used for the evaluation of heart function in the laboratory because of the ease, speed, and high success rate that accompanies this model^22^,^23^,^24^,^25^,^26^,^27^. Hearts transplanted by heterotopic cervical heart transplantation have been assessed for heart rate, heart weight, electrocardiography, and histopathology^22^,^23^,^28^. The addition of PET analysis offers several advantages, including a higher sensitivity and specificity, as well as the ability to measure myocardial blood flow and myocardial metabolism in absolute terms^25^. The most commonly used PET tracer is [^18^F]-FDG^29^,^30^. The dependence of the myocardium on glucose metabolism makes [^18^F]-FDG an ideal tracer in this setting^29^,^30^. In the present study, we applied FDG-PET to evaluate heart function after heterotopic cervical heart transplantation by comparing donor and recipient hearts. The results of the FDG-PET analysis showed a similar pattern at 90 min and 7 days in each group (Fig. 5). In addition, the post-transplantation heart rate, weight on day 100, and histopathological findings (TUNEL staining) at 90 min showed patterns similar to the results of the FDG-PET analysis in each group (Figs 4 and 5). These results indicated that there were no significant differences in transplanted heart function between the PC and CO24h groups. Furthermore, high-pressure preservation in a mixture of CO and O_2_ gases is better for reducing IRI in donor hearts compared with the simple immersion method using the UW solution. In this study, inbred rats were used to confirm all of the effects of high-pressure gas preservation in rat hearts except for the rejection response. Immunologic evaluation of the allogeneic transplantation with this preservation method is now in progress.

In the clinic, organ preservation using static cold storage has been used. Recently, machine perfusion preservation for donor hearts was clinically established and drew attention. Machine perfusion was shown to be non-inferior compared to the static cold storage for 4 h, which had excellent clinical performance and enabled the successful recovery and transplantation of hearts from high-risk, brain-dead donors and circulatory death donors^6^. The high-pressure gas preservation can be classified as a cold storage method. The present study demonstrated our preservation method was safe and could maintain the quality of the rat donor heart for 24 h. We believe that high-pressure gas preservation can be made available in the clinic. To obtain detailed insight regarding the function of the preserved heart, including long-term prognosis after transplantation, studies using an orthotopic transplant model in a large animal such as a pig or monkey is needed.

## Conclusion

The high-pressure preservation of organs in a mixture of CO and O_2_ gases has the potential to preserve the quality of hearts for organ transplantation. There have been many reports of several medical gases that have organ- and/or cell-protective effects. Specifically, in addition to CO, nitric oxide, hydrogen, hydrogen sulfide, xenon, and ozone have been reported to show these effects^31^. The high-pressure preservation method has the advantage that organs can be kept in a chamber filled with a high-pressure mixture of gases without any special solutions or machines. High-pressure gas preservation using a combination of gases under the appropriate pressure may clinically contribute to enhanced operative outcomes following organ transplantation.

## Methods

### Animals

This experiment used an inbred line of LEW/SsN Slc rats (male, 10-weeks old, average weight: 230 g, range: 220–245 g, donors: n = 88, recipients: n = 88) were purchased from the Shizuoka Laboratory Animal Center (Shizuoka, Japan). All of the handling and care of the rats conformed to the National Institutes of Health (NIH) guidelines for animal research, and all of the experimental protocols involving animals were approved by the National Research Institute for Child Health and Development Animal Care and Use Committee (Permit Number: 254). All of the experiments involving animals were performed in accordance with these guidelines and experimental protocols. All efforts were made to minimize animal suffering.

### Experimental design

The heart was extracted from the rat under deep anesthesia using pentobarbital (50 mg/kg, Kyoritsu Seiyaku Corporation, Tokyo, Japan). Then, the blood was removed using Krebs-Henseleit solution following an aortic/pulmonary artery incision, and the solution was further infused. A custom-built 7 air pressure-resistant chamber (L: 165 mm/W: 165 mm/H: 200 mm, material: iron, Nakamura Iron Works Co., Ltd. Tokyo, Japan) was cooled to 4 °C in advance. Next, the rat hearts were preserved in the chamber at 4 °C under each condition as follows: (i): the hearts were hung inside a chamber that was filled with a mixture of CO and O2 gases (7000 hPa; PCO = 4000 hPa + PO2 = 3000 hPa), and a flask with 50 ml of distilled H2O was placed within the chamber to maintain humidity for 24 h (CO24h group: n = 19) or 48 h (CO48h group: n = 16). (ii): the hearts were hung inside a chamber that was filled with room air (1000 hPa), and a flask with 50 ml of distilled H2O was placed within the chamber to maintain humidity for 24 h (Air24h group: n = 4) or 48 h (Air48h group: n = 4). (iii): the hearts were completely immersed in a UW solution (Viaspan, Du Pont, Wilmington, DE, USA) and then placed in a chamber filled with room air (1000 hPa) for 24 h (UW24h group: n = 12) or 48 h (UW48h group: n = 6). (iv): the hearts were immersed in extracellular fluid containing 5% glucose (Lactec D, Otsuka Pharmaceutical Co. Ltd., Tokyo, Japan; Na 130 mM, K 4 mM, Ca 3 mM, Cl 109 mM, lactate 28 mM, glucose 5%) and then placed in a chamber filled with room air (1000 hPa) for 24 h (EF24h group: n = 4) or 48 h (EF48h group: n = 4). Rat hearts used immediately after extraction served as the positive control group (PC group, n = 19). Our previous study revealed that rat hearts cannot be resuscitated after 48 hours of preservation under the conditions of P helium (He) = 4000 hPa + PO2 = 3000 hPa (11). Therefore, we considered that the Air24h, Air48h, UW24h, UW48h, EF24h, and EF48h groups were the negative controls for the high-pressure preservation method that used a mixture of CO and O2. The preserved hearts were removed from the pressure-resistant chamber, and heterotopic cervical heart transplantation to a recipient rat was performed.

### Short-term evaluation (post-transplantation at 90 min)

After 90 min, the post-transplanted hearts were removed from recipient rats under anesthesia and were processed for histological analysis. COHb levels in the recipient’s arterial blood ware measured to determine whether the dissolved CO in the preserved heart affected the recipient (n = 6). The preserved hearts were functionally evaluated 90 min after transplantation when ischemia reperfusion injuries are most severe. The size of the myocardial infarction was macroscopic estimated using 2,3,5-triphenyltetrazolium chloride (TTC) staining (n = 12). The structure and apoptosis of the grafts were confirmed using HE and TUNEL staining, respectively (n = 12). In addition, for [^18^F]-fluorodeoxyglucose positron emission tomography (FDG-PET) analysis, heterotopic heart transplantation from the three groups (PC group: n = 8, CO24h group: n = 8, and CO48h group: n = 8) was performed, and then, the post-transplanted hearts (PC group: n = 4, CO24h group: n = 4 and CO48h group: n = 4) were evaluated by FDG-PET at 90 min.

### Long-term evaluation (post-transplantation at 100 days)

To evaluate revival and survival rates after transplantation, we observed the subcutaneous pulsation of the post-transplanted heart in the neck daily, and the heart rates for each group (PC group: n = 6, CO24h group: n = 6, CO48h group: n = 6, Air24h group: n = 4, Air48h group: n = 4, UW24h group: n = 6, UW48h group: n = 6, EF24 group: n = 4, and EF48 group: n = 4) were measured by electrocardiogram (ECG) at 100 days after transplantation. The post-transplanted hearts that survived 100 days were removed from the recipient rats under anesthesia, and the heart weight was recorded. To investigate the histopathology of post-transplanted hearts after high-pressure preservation in a mixture of CO and O2 gas, heterotopic heart transplantation from the three groups (PC group: n = 4, CO24h group: n = 4, and CO48h group: n = 4) was performed.

### Evaluation of the preserved rat hearts by heterotopic cervical heart transplantation

Recipient rats (male, 10 weeks old: n = 106) were deeply anesthetized with pentobarbital (65 mg/kg body weight) and then kept under anesthesia using isoflurane (ISOFLU^®^, Dainippon Sumitomo Pharma Co., Ltd.) in the inhalation anesthesia apparatus (Univentor 400 Anesthesia Unit, Univentor Ltd.). The rats were anesthetized with isoflurane by mask ventilation (air 400–500 ml/min, isoflurane 1.5–2.0%) (Univentor 400 anesthesia Unit; Univentor, Ltd., Zejtun, Malta). Following transplantation, the skin was sutured after the heart rate stabilized. The pulsation of the heart was observed to be palpating in the neck, and the post-transplant hearts were examined by ECG. The absence of a waveform for 15 min was considered to be cardiac arrest.

### Measurement of CO concentration in the preservation chamber

CO concentration was monitored with a CO detective instrument (B&K Precision Corp., California, USA). In opening the valve of the chamber after preservation, it was monitored at two points: a - (human working area at 30 cm from the chamber) outside the fume hood and b - (10 cm directly above the chamber) inside the fume hood (Fig. 2A). Furthermore, it was measured 10 cm directly above the preserved heart, which was taken from the chamber.

### Assessment of COHb by blood gas analysis

Arterial blood was obtained from naive LEW rats and CO-treated recipients at 90 min and 24 h after heart transplantation. COHb was analyzed with a blood gas analyzer (Siemens RAPIDPoint 405, Germany).

### Estimation of myocardial infarct size

The size of the myocardial infarction in the 4 groups was estimated by 2,3,5-triphenyltetrazolium chloride (TTC) staining. Briefly, after reperfusion, the hearts were weighed and cut into 2-mm-thick slices vertical to the atrioventricular groove. The slices were stained by incubation in 2% TTC solution in phosphate buffer (0.1 M) at 37 °C for 15 min and then fixed in 4% paraformaldehyde solution. Infarct size (unstained by TTC) was measured by planimetry using the Image Pro Plus 5.0 software (Media Cybernetics, Silver Spring, MD, USA) and was expressed as a percentage of the total heart.

### Light microscopy

Rat hearts from each group were fixed in Bouin’s solution for three hours. The samples were then washed, dehydrated with an ethanol series, and embedded in paraffin. Serial 6-μm sections were cut with a microtome and stained with Gill’s hematoxylin III and 2% eosin Y.

### TdT-mediated dUTP nick end labeling (TUNEL) staining

Apoptosis of the myocardial cells was detected using the *in situ* TUNEL assay (Cardio TACS *In Situ* Apoptosis Detection Kit; Trevigen, Gaithersburg, MD, USA) according to the manufacturer’s instructions. For statistical analysis, the sections were analyzed at 100-times magnification by light microscopy. The number of TUNEL-positive cells was counted in 20 areas of 1 mm^2^ that were randomly chosen.

### FDG-PET

To accelerate the uptake of [^18^F]-FDG, the recipient rats from each group were intraperitoneally injected with a single dose of glucose (1 mg/g body weight) and insulin (8 mU/g body weight). After 30 min, a single dose of [^18^F]-FDG (37 MBq; Nihon medi-physics Co., Ltd, Tokyo, Japan) was administered via the tail vein. After 60 min, the rats from each group were analyzed by PET imaging (Inveon, Siemens AG, Germany). A semiquantitative evaluation was performed by calculating the maximum accumulation of [^18^F]-FDG in the recipient heart relative to that of the donor heart.

### Electrocardiogram (ECG)

The basic limb lead method was slightly modified using a multi-factor electrocardiograph (Power Lab ML826; AD Instruments Japan, Inc., Nagoya) under inhaled ether anesthesia. Two electrodes were diagonally attached to the upper right or lower left sides of the recipient or the transplanted donor hearts, respectively. To measure the electric waves from both the recipient and the transplanted donor hearts at the same time, two electrodes were diagonally attached to the upper right side of the transplanted donor heart and the lower left side of the recipient heart. The evaluation was based on the presence of a regular waveform appearing at ECG lead II.

### Statistical analysis

The values are presented as the means ± standard deviations. The significance of the differences was determined using the ANOVA/post-hoc tests. P < 0.05 was considered statistically significant.

## Additional Information

**How to cite this article**: Hatayama, N. *et al*. Functional evaluation of rat hearts transplanted after preservation in a high-pressure gaseous mixture of carbon monoxide and oxygen. *Sci. Rep.* **6**, 32120; doi: 10.1038/srep32120 (2016).

## Figures and Tables

**Figure 1:**
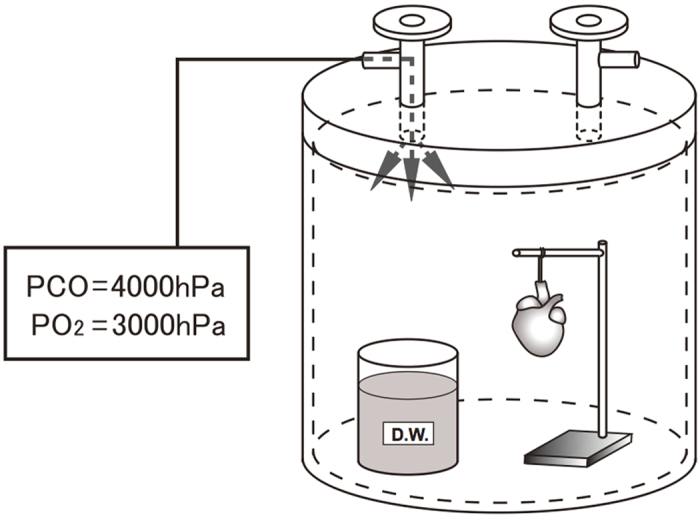
A schematic of the preservation method. The chamber is filled with PCO (4000 hPa) and PO_2_ (3000 hPa). During the heart preservation, a flask with 50 ml of distilled water is placed in the chamber to maintain humidity. Threads are tied to the arteries of the heart, which is suspended with a wire.

**Table 1:** Revival rate immediately after transplantation and survival rate after 100 days for each group.

Group	Preservation method	PT(h)	n	RR (%)	SR (%)	SDs
PC	—	0	6	6/6(100)	5/6 (83)	83, >100 × 5
C024h	PCO = 4000hPa + PO_2_ = 3000hPa	24	6	6/6(100)	5/6(83)	74, >100 × 5
C048h	PCO = 4000hPa + PO_2_ = 3000hPa	48	6	6/6(100)	4/6 (67)	14, 76, >100 × 4
Air24h	Air	24	4	0/4 (0)	0/4 (0)	0 × 4
Air48h	Air	48	4	0/4 (0)	0/4 (0)	0 × 4
UW24h	University of Wisconsin solution	24	6	1/6(16.7)	0/6 (0)	13, 0 × 5
UW48h	University of Wisconsin solution	48	6	0/6 (0)	0/6 (0)	0 × 6
EF24h	Extracellular fluid with 5% glucose	24	4	0/4 (0)	0/4 (0)	0 × 4
EF48h	Extracellular fluid with 5% glucose	48	4	0/4 (0)	0/4 (0)	0 × 4

PT: Preservation time, RR: Revival rate; immediately after transplantation, SR: survival rate, after 100 days, SDs: Survival days
